# Supplementary material for: Quantitative Comparison of 3D-1D Vascular Coupling Models: Lateral Average versus Sphere of Influence Methods
Source: bioRxiv. 2026 Jul 16:2026.07.12.738114. Preprint. [Version 1] doi: 10.64898/2026.07.12.738114 (PMC13404840; doi:10.64898/2026.07.12.738114)
Supplement: Supplementary file 1 [file NIHPP2026.07.12.738114v1-supplement-1.pdf]

bioRxiv preprint doi: https://doi.org/10.64898/2026.07.12.738114; this version posted July 17, 2026. The copyright holder for this preprint (which was not certified by peer review) is the author/funder, who has granted bioRxiv a license to display the preprint in perpetuity. It is made available under a CC-BY 4.0 International license.

Supporting information

S1 Fig. Coordinates of each graph node and radii of each vessel.

Point	Coordinates
A	(0,20,15)
B	(10,20,15)
C	(22,13,15)
D	(22,28,15)
E	(15,5,15)
F	(15,35,15)
G	(38,5,15)
H	(38,35,15)

Segment	Radius
AB	4mm
BC	3mm
BD	3mm
CE	2mm
CG	3mm
DF	2mm
DH	3mm

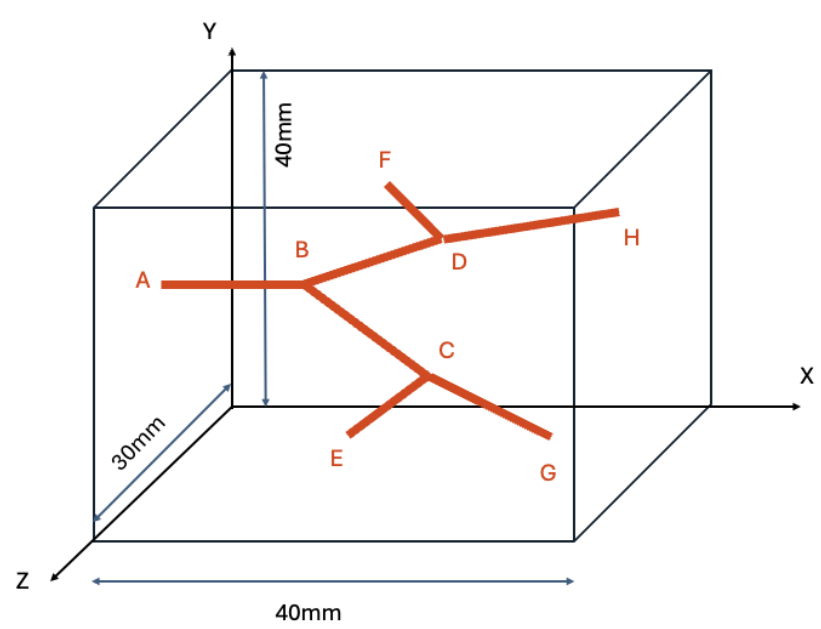

Fig 16. Coordinates of each graph node and radii of each vessel
